# An exploration of the influence of animal and object categories on recall of item location following an incidental learning task

**DOI:** 10.1177/17470218241238737

**Published:** 2024-03-21

**Authors:** Dan PA Clark, Nick Donnelly

**Affiliations:** Department of Psychology, Liverpool Hope University, Liverpool, UK

**Keywords:** Object location, object based attention, memory for object location, attention

## Abstract

The current study explores the role of attention in location memory for animals and objects. Participants completed an incidental learning task where they rated animals and objects with regard to either their ease of collection to win a scavenger hunt (Experiments 1a and b) or their distance from the centre of the computer screen (Experiment 2). The images of animals and objects were pseudo-randomly positioned on the screen in both experiments. After completing the incidental learning task (and a reverse counting distractor task), participants were then given a surprise location memory recall task. In the location memory recall task, items were shown in the centre of the screen and participants used the mouse to indicate the position the item had been shown during the incidental encoding task. The results of both experiments show that location memory for objects was more accurate than for animals. While we cannot definitively identify the mechanism responsible for the difference in the location memory of objects and animals, we propose that differences in the influence of object-based attention at encoding affect location memory when tested at recall.

## Introduction

The encoding and subsequent recall of object location have been extensively studied (e.g., Barel, 2018; Brockmole & Wang, 2002; Huttenlocher et al., 1991; Lansdale, 1998; Lansdale et al., 2013; Naveh-Benjamin, 1987, 1988; Schutte et al., 2003). Initially, Hasher and Zacks (1979) argued that location was encoded automatically (i.e., without conscious intention and requiring minimal cognitive resources), but more recent work supports a more nuanced view. In particular, object location memory is better when attention can be focused on items at encoding (Postma & De Haan, 1996), is subject to bias from rewards that influence the allocation of focused attention at encoding, and is better in those individuals with a heightened ability to sustain attention over time (Barel & Tzischinsky, 2020).

The evidence shows that the ability to focus and sustain attention over the time course of encoding influences the accuracy of object location memory at recall. In the present study, we extend this exploration of how visual attention influences location memory by examining location memory for animals and objects. A number of studies have reported that animals engage attention differently to objects (e.g., New et al., 2007; Öhman et al., 2001; Tipples et al., 2002). To take one example, Guerrero and Calvillo (2016) had participants try to detect and name two target items (framed by a red border) set among a series of 15 items in a rapid serial visual presentation (RSVP) of a set of images. The results showed that the identification of animals in an RSVP stream was better than that of objects. Guerrero and Calvillo concluded that animals attracted attention more readily than objects.

**Figure 1.**
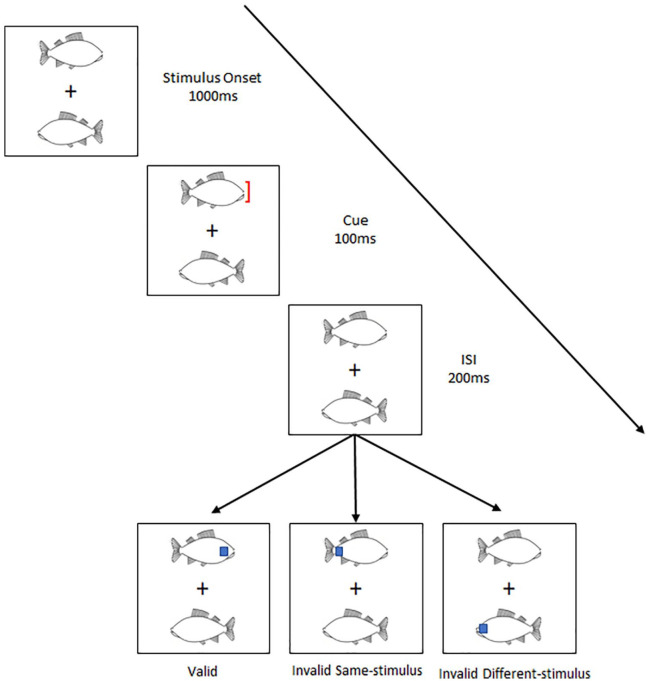
A schematic representation of Hu et al.’s (2023) experimental paradigm.

Hu et al. (2023) explored a related issue: whether animals and objects differ in the kind of visual attention that they attract. Using a modified version of the two-rectangles paradigm (Egly et al., 1994), Hu et al. explored the extent to which animals and objects attract object-based attention (see Figure 1 for a schematic of their procedure). In their study, participants were shown a pair of identical items presented for 1.1 s on either side of a fixation cross. These items could either be a chicken or a fish (i.e., animal), or a key or a shuttlecock (i.e., objects).

In the final 100 ms of item presentation, an exogenous cue (a red bracket) was presented on the tip of one of the items. After a 200-ms interstimulus interval (ISI), participants were asked to perform a go–no-go task to the appearance of a blue square. The blue square might appear at the same location as the cue, at a different location but on the same item as the cue, or at the location of the other item. The critical result reported by Hu et al. was that participants were quicker to respond to the blue square when it appeared at an uncued location on the same than different item. However, this was significantly quicker when the initial items presented were animals. Hu et al. suggest that their results demonstrate that animals more readily attract object-based attention than objects. The present study builds on this finding.

Evidence suggests that there are two forms of object-based attentional selection (Matsukura & Vecera, 2009, 2011; Vecera & Farah, 1994): objects can be selected either early or late. When selected early, objects are represented as a grouped array of features such that spatial location is encoded with respect to an environmental reference frame (Lavie & Driver, 1996; Matsukura & Vecera, 2011). When selected late, objects are represented as a single, spatially invariant, item (Matsukura & Vecera, 2009) using an object-centred reference frame (see Scholl, 2001). Our proposal is that differences in the allocation of object-based attention will impact the accuracy of the later recall of the spatial location of objects by virtue of differences in the reference frame used when encoding objects. Here, we make the minimal assumption that the accuracy of object location at recall will be impacted if an environmental reference frame was not prioritised at encoding.

As far as we can ascertain, only a single study has explored if a difference exists in location memory for animals and objects, and this was with respect to words describing animals and objects and not images (Gelin et al., 2018).^1^ In Gelin et al.’s (2018) study, 64 words (32 words naming animate items and 32 naming objects) were presented in one of four quadrants on the screen. During an incidental encoding phase, participants were instructed to indicate if the words shown related to animate or inanimate items. In a subsequent recognition test, participants were presented with the 64 words shown at encoding along with 64 lures. Participants were asked to indicate if they had seen each word at encoding and, if they responded positively, to report the quadrant they believed the word to have been. The results showed that the recall of the location of words naming animals were better recalled than those of objects. It is unclear, however, how the findings of Gelin et al. (2018) help to establish a clear prediction for the present study. This is because words are a very different class of visual stimulus to pictorial images.

In the absence of clear prior relevant data, the findings of Guerrero and Calvillo (2016) and Hu et al. (2023) suggest two possible outcomes for location memory of differential attention to animals and objects at encoding. First, if animals engage early object-based attention more readily than objects, then their location may be better recalled in a later retrieval test. If this is the case, then the average Euclidean distance of the deviation of location memory judgements (relative to the true position shown at encoding) should be lower for images of animals than images of objects. However, if animals engage late object-based attention more readily than objects, then memory for their precise spatial location may be relatively diminished when tested in a later retrieval test. If the memory for their precise spatial location is relatively diminished, then we predict that the standard deviation of location judgements at recall for animals will show an increased variability across a set of trials relative to those objects.

In the present experiments, participants were asked to recall the location of images of animals and objects originally presented across a set of encoding trials. The position of the items at encoding was pseudo-randomly manipulated and participants performed a cued object location memory task. We hypothesise that the accuracy, and deviation, of location memory judgements for animals will differ from that of objects. In the present study, we do not explore the allocation of attention to either stimulus group directly, but rather the impact of presumed differences in attentional allocation to objects and animals on location memory. If animals are selected using late object-based attention but objects are selected using early object-based attention, then we hypothesise that the location memory for animals will be less accurate than for objects.

## Experiment 1a

### Method

#### Transparency and openness

We report how we determined our sample size, all manipulations, and all measures in the present series of experiments and follow Journal Article Reporting Standards (Kazak, 2018). All processed data, analysis code, and research materials are available at https://osf.io/uz9rk/. Data were analysed using R, version 4.2.2 (R Core Team, 2022) and the package *ggplot2*, version 3.4.9 (Wickham, 2016). The study design and analysis were not preregistered.

### Participants

Thirty-seven (7M: 30F) participants with a mean age of 20.65 (*SD* = 3.60) took part in the current study. All participants were students at Liverpool Hope University. The sample size was determined a priori by G*Power 3.1.9.7 (Faul et al., 2007) using the following parameters: alpha = .05, power = .80, and η^2^_p_ = .04. It was estimated that a sample size of 36 was required to achieve sufficient power to detect the effect. The sample size was estimated using Gelin et al. (2018) and was estimated using the effect size for the main effect of Stimuli (Animacy) in a 2 × 2 repeated measures design.^2^

### Design

The study had a 2 × 2 repeated measures design with two within participants IV’s: Stimulus (with two levels, Animals and Objects) and Distance (with two levels, Near and Far). The dependent variable in the study was the deviation from target location, measured in pixels.

### Materials

**Figure 2.**
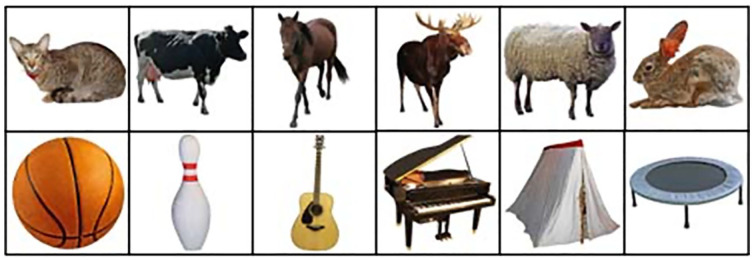
The target animals and objects used in the current study.

The stimuli used in the current study were all images taken from the Bank of Standardised Stimuli (BOSS – Brodeur et al., 2014). They included 12 images (see Figure 2): 6 animals (cat, sheep, rabbit, cow, moose, horse) and 6 objects (guitar, bowling pin, basketball, tent, trampoline, and a piano). The BOSS stimuli database was used as it contains 930 colour photographs depicting a wide range of items. In addition, the BOSS database provides recent (2014) normative data which allowed the stimulus images to be controlled along a series of dimensions (familiarity, visual complexity, object agreement, viewpoint, and manipulability). Table 1 shows the mean score for each dimension and also the analysis comparing the stimuli.

**Table 1.** The mean and standard deviation each controlled the dimension and comparison analysis. All scores were taken from the BOSS Stimuli database normative data (Brodeur et al., 2014).

Condition	Familiarity	Visual complexity	Object agreement	Viewpoint agreement	Manipulability
Animals	4.42 (0.22)	2.92 (0.07)	3.85 (0.30)	3.69 (0.30)	2.54 (0.49)
Objects	4.51 (0.14)	2.26 (0.70)	3.96 (0.46)	4.03 (0.24)	4.00 (0.73)
Comparison	*t*(10) = −0.85, *p* = .42	*t*(5.09) = 2.67, *p* = .07	*t*(10) = −0.47, *p* = .65	*t*(10) = −2.15, *p* = .06	*t*(10) = −4.08, *p* = .002

Table 1 shows that there is only a significant difference between the animals and objects stimulus sets for the manipulability dimension. This is not particularly surprising given that by definition, animals are living and capable of independent decisions and movement.

As Experiment 1a was an incidental learning task, a cover task was used to ensure participants focused on the relevant parts of the scene without overtly indicating that memory would be tested. The encoding task employed was a Scavenger Hunt Task, which was adapted from previous studies where it has been used to test location memory (Clark & Bruno, 2016; Nairne et al., 2012). The scavenger hunt encoding scenario can be seen below.

#### Scavenger hunt encoding scenario

*In this task, we would like you to imagine that you have been invited to take part in a scavenger hunt. Your goal is to win this contest and as such, you will need to collect as many of the target items as possible. We are going to show you pictures of these items that will be displayed in various locations on the screen and we would like you to rate how easy it is to collect. You should consider your initial position is located at the centre of the screen (indicated by a cross) and you should take each item’s distance from the starting point and ease of collection (e.g. items further from the starting point will take longer to collect and items that move will need to be captured) into account when making your rating.*

*Remember, your goal is to collect as many items as possible to help you win the contest.*

All target items were 80 × 80 pixels in size and were displayed on a 43-in. monitor with a screen resolution of 1,920 × 1,080. Participants were positioned approximately 90 cm from the centre of the screen. Target item’s locations were pseudo randomised and were displayed either Near (within 375 pixels) to the centre of the screen (where a fixation cross was displayed) or Far (over 375 pixels) from the centre of the screen. The mean distance from the centre of the screen was comparable for both stimulus conditions: Animals 499.98 (±244.23) and Objects 492.63 (±273.21) pixels. This manipulation allowed for a check that the encoding task had been completed correctly (since following the scenario of near items should be easier to collect than far items). All targets were displayed in their own discrete location, with no overlap between targets.

### Procedure

The experimental procedure replicated that of Clark and Bruno (2016) and was divided into three sections: Encoding, Distractor, and Retrieval.

#### Encoding and distractor tasks

After participants had provided informed consent, they were asked to read the scavenger hunt scenario. They then viewed each object in turn (in a randomised order) and were asked to rate the ease of collection from 1 (*very difficult*) to 9 (*very easy*). Each item was displayed for 5 s and then they were asked to complete the rating on a screen which only included the rating scale. Once participants had rated all of the target items, they were asked to complete a distractor task. This task was to reverse count in multiples of 3 backwards from 999 for 30 s.

#### Retrieval

In the retrieval task, participants were cued with a picture of each target item and then on the next screen (a blank screen with a cross at the centre) they were asked to indicate where they recalled that target having been displayed during the encoding rating task by clicking the mouse in the recalled location. The targets were cued at random and once each location had been indicated, participants were thanked for participation and debriefed.

### Results

The results were separated into two sections, the encoding ratings and the retrieval data.

#### Encoding ratings

The first stage of the analysis was to explore if there was a difference in the encoding ratings and encoding decision times. It was predicted that, following the encoding task, items that were positioned further away and were more complicated to collect would be rated as more difficult to collect (see Table 2 for descriptive statistics).

**Table 2.** The mean (*SD*) encoding ratings and response times (s) in Experiment 1a for animals and objects at both the near and far distances.

Distance	Encoding ratings		Encoding decision time (s*)*	
	Animals	Objects	Animals	Objects
Near	5.83 (2.20)	7.03 (1.63)	2.82 (1.63)	1.82 (1.04)
Far	4.57 (2.33)	4.91 (2.08)	2.75 (1.35)	1.90 (9.49)

When exploring the distributions of these data, we found a number of outliers. To control for this, we conducted a reciprocal transformation^3^ on the data and used these in the statistical analysis. To test for differences in the encoding ratings, a 2 × 2 repeated measures ANOVA, with Stimulus (two levels, Animals and Objects) and Distance (two levels, Near and Far) was conducted. This revealed a significant effect of Stimulus, *F*(1, 36) = 10.25, MSE = 2.32, *p* = .003, η^2^_p_  = .22, a significant effect of Distance: Distance *F*(1, 36) = 37.46, MSE = 3.08, *p* < .001, η^2^_p_  = .51, and a significant interaction between Stimulus and Distance, *F*(1, 36) = 10.16, MSE = 0.8, *p* = .003, η^2^_p_  = .22. Further exploration of the significant interaction using Bonferroni corrected pairwise comparisons revealed a significant effect of Stimuli at the Near distance, *t*(36) = −4.75, *p* < .001, *d* = −1.27, but not Far distance, *t*(32) = −1.07, *p* = .292, *d* = −0.33. The analysis also showed a significant effect of Distance in the Animals, *t*(36) = 4.28, *p* < .001, *d* = 1.30, and Objects, *t*(6.51), *p* < .001, *d* = 2.23.

To explore the encoding decision time data, the 2 × 2 repeated measures ANOVA was repeated changing the dependent variable from encoding ratings to encoding decision time. Again, these data contained outliers, so a reciprocal transformation was used. This analysis revealed a main effect of Stimulus, *F*(1, 36) = 11.58, MSE = 0.15, *p* = .002, η^2^_p_  = .24 but no significant effect of Distance, *F*(1, 36) = 0.33, MSE = 0.15, *p* = .571, η^2^_p_  < .01 and no significant Stimulus × Distance interaction, *F*(1, 36) = 0.33, MSE = 0.04, *p* = .570, η^2^_p_  < .01.

Together, these findings suggest that the encoding task was well engaged with, since targets that were further away were rated as harder to collect. The decision time data support this as animals were considered by participants for longer than objects.

#### Retrieval

The first stage of the analysis was to calculate the distance between the original and recalled target locations. This procedure was replicated from previous studies exploring retrieval of object location memory (Clark & Bruno, 2016; Nairne et al., 2012) and used Euclidean distance to estimate the deviation scores, calculated as

$$\sqrt{{\left(X_{1}-X_{2}\right)}^{2}+{\left(Y_{1}-Y_{2}\right)}^{2}}$$

where *X*_1_ and *Y*_1_ represent the target location at encoding and *X*_2_, *Y*_2_ at retrieval. The deviation scores represent accuracy where zero represents perfect recall of location and larger scores represent a further deviation from the target location.

Once we had calculated the Euclidean distances for each individual target, we used these data to calculate a mean recall and standard deviation of both animals and objects for each participant.

The data were analysed in three ANOVAs with the factors of Stimulus (within participants with two levels, Animals and Objects) and Distance (within participants with two Levels, Near and Far), with separate analyses being run on the mean accuracy of recall, standard deviation of accuracy, and decision time to recall the target location. The mean accuracy of recall represents the mean recalled distance from the location at the centre of the target location at encoding (defined as the centre of the 80 × 80 pixel virtual box in which all stimuli were placed). We also explored the standard deviation of accuracy. It may be that the centre of the virtual box does not accord with the perceived centre of an object. It might be, for example, that the perceived centre of an animal is shifted towards its head. To take account of this fact, the standard deviation measure provides a measure of consistency of the recall of target objects location. To make the measures easier to distinguish, in the remainder of the article we refer to mean accuracy of the report of location from the centre of the virtual box surrounding each target as the mean Euclidean distance error, and the *SD* report of location as the consistency of Euclidean distance error and decision time as retrieval decision time.

**Figure 3.**
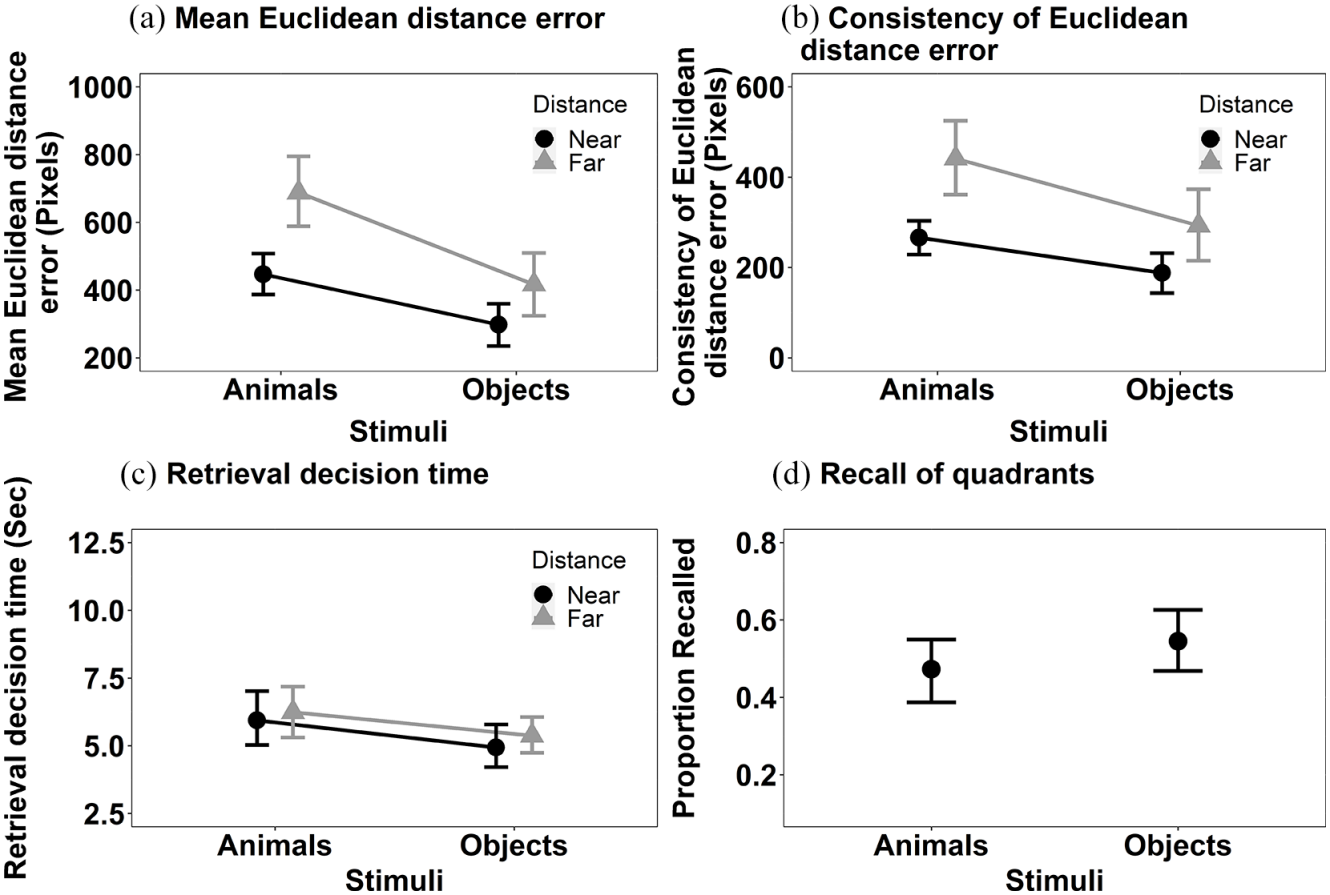
The mean recall performance in Experiment 1a for (a) mean Euclidean distance error for animals and objects in both near and far locations; (b) consistency of Euclidean distance error for animals and objects in both near and far locations; (c) the retrieval decision time for animals and objects in both near and far locations; and (d) proportion of targets correctly recalled in the displayed quadrant. All error bars represent ±95% CI.

*Mean Euclidean distance error* (Figure 3a): The main effects of Stimulus, *F*(1, 36) = 27.79, MSE = < 0.001, *p* < .001, η^2^_p_  = .44, and Distance, *F*(1, 36) = 11.31, MSE < 0.001, *p* = .002, η^2^_p_  = .24, were significant. Objects were recalled more accurately than animals and items presented near to the centre of the screen at encoding were recalled more accurately than those items displayed further away. The interaction between Stimulus and Distance, *F*(1, 36) = 6.97, MSE < 0.001, *p* = .012, η^2^_p_  = .16, was also significant. Further exploration of the significant interaction using Bonferroni corrected pairwise comparisons revealed a significant effect of Distance with both objects, *t*(36) = 3.28, *p* = .002, *p* < .001, *d* = .002, and animals, *t*(36) = 2.27, *p* = .029, *d* = .001, but the numerical magnitude of the effects is bigger with animals than objects.

*Consistency of Euclidean distance error* (see Figure 3b): The main effects of Stimulus *F*(1, 36) = 13.54, MSE = 34,979, *p* < .001, η^2^_p_  = .27 and Distance, *F*(1, 36) = 17.18, MSE = 42,258, *p* < .001, η^2^_p_ = .32, both reached significance. The consistency of Euclidean distance error recall was smaller for objects than animals. The interaction between Stimulus and Distance, *F*(1, 36) = 1.79, MSE = 25,942, *p* = .190, η^2^_p_  = .05, did not reach significance.

*Retrieval decision time* (see Figure 3c): The retrieval decision time data were transformed using a reciprocal transformation, prior to data analysis. The main effect of Stimulus reached significance, *F*(1, 36) = 8.02, MSE = 0.005, *p* = .008,η^2^_p_ = .18, but Distance did not, *F*(1, 36) = 2.46, MSE = 0.005, *p* = .125, η^2^_p_ = .06. Decisions for objects were made faster than those for animals. The interaction between Stimulus and Distance did not reach significance, *F*(1, 36) = 0.48, MSE = 0.006, *p* = .493, η^2^_p_  = .01.

We also conducted an additional analysis. The primary analysis considered accuracy in recall of spatial location using a coordinate Euclidean distance measure of deviation. A commonly conducted analysis of spatial location memory is with respect to correctly recalling the quadrant of the screen in which an object was first presented to allow a formal comparison to other studies (e.g., Gelin et al., 2018) that use this measure of spatial location memory. We supplemented the analysis presented above, where a coordinate measure of distance was estimated using Euclidean distance, with a categorical one. To do this, the screen was divided into four equally sized quadrants and the proportion of targets correctly recalled as being in those quadrants was computed (note that dividing the screen into quadrants does not allow a meaningful comparison of distance to be made). These data are shown in Figure 3d and were analysed using a Wilcoxon test (as these data did not meet the parametric assumptions [Shapiro–Wilk for the Animal recall was significant, *p* = .039]). The effect of Stimulus was significant, V = 107.5, *p* = .03, *r* = .36. Consistent with the primary analyses, the proportion of targets recalled in the correct quadrant was higher for objects than for animals.

### Discussion

The results of Experiment 1a show that location memory was more consistent and accurate for objects than animals. The results with respect to the accuracy of location memory held across coordinate and categorical measures of recall. The results are consistent as hypothesised in the “Introduction” that if the location memory for animals is impacted by late (spatially invariant) object-based attention, then we predict that location memory will show increased variability with animals than objects.

One potential account of the consistency of Euclidean distance error and mean Euclidean distance error results is that participants were less conservative in their decision making to animals than objects. However, the decision time data at encoding and retrieval do not support this interpretation. The scavenger hunt decision at encoding and the retrieval of the locations were made more slowly for animals than objects. In other words, not only was the spatial location of animals recalled less accurately than those of objects, participants took longer to encode and reach decisions about animals than objects.

Despite predicting the results of Experiment 1 in relation to the consistency of Euclidean distance error of recall, we were surprised by the findings in relation to mean Euclidean distance error and retrieval decision time. The dual findings of lower accuracy allied to increased variation in location recall suggests that the recall of the locations of animals were not just more widely distributed around the true original location, but were subject to a higher deviation from the original target location. Likewise, the effect of stimulus on decision time suggests that the poor recall of the location of animals did not occur by virtue of reduced encoding or retrieval effort. Given the fact that the results of Experiment 1a were more striking than we expected, we decided to seek confirmatory evidence in a direct replication of Experiment 1a before proceeding.

## Experiment 1b

Experiment 1b was a direct replication of Experiment 1a, as such, the design, materials, and procedures and analysis were exactly the same. The only difference was that the experiment was conducted using a new naive set of participants.

### Participants

Thirty-five (7M: 28F) participants with a mean age of 23.40 (*SD* = 8.01) took part in the current study. All participants were staff or students at Liverpool Hope University. The sample size was calculated for Experiment 1a.

### Results

The data were analysed as in Experiment 1a.

#### Encoding

The mean encoding ratings and encoding decision time for each condition is shown in Table 3. With respect to encoding ratings, the main effects of Stimulus, *F*(1, 34) = 13.24, MSE = 0.009, *p* < .001, η^2^_p_  = .28, and Distance, *F*(1, 34) = 15.84, MSE = 0.005, *p* < .001, η^2^_p_  = .32, were significant. Items appearing near the centre were rated easier to collect. Animals were rated more difficult to collect than objects. The main effects of Stimulus and Distance did not interact, *F*(1, 34) = 0.89, MSE = 0.026, *p* = .352 η^2^_p_  = .03.

**Table 3.** The mean (*SD*) encoding ratings and encoding decision times (s) in Experiment 1b for animals and objects at both the near and far distances.

Distance	Encoding ratings		Encoding decision time (s)	
	Animals	Objects	Animals	Objects
Near	6.24 (2.14)	7.43 (1.38)	3.83 (2.17)	2.18 (1.27)
Far	4.83 (2.00)	6.10 (1.71)	3.65 (2.06)	2.34 (1.44)

With respect to encoding decision time, the main effect of Stimulus was significant, *F*(1, 34) = 27.91, MSE = 0.09 *p* < .001, η^2^_p_  = .45. Decisions were made faster with objects than animals. Neither, the main effect of Distance, *F*(1, 34) = 0.21, MSE = 0.03, *p* = .647, η^2^_p_  < .01, nor the interaction between Stimulus and Distance, *F*(1, 34) = 0.18, MSE = 0.03, *p* = .675, η^2^_p_  < .01, reached significance.

#### Retrieval

**Figure 4.**
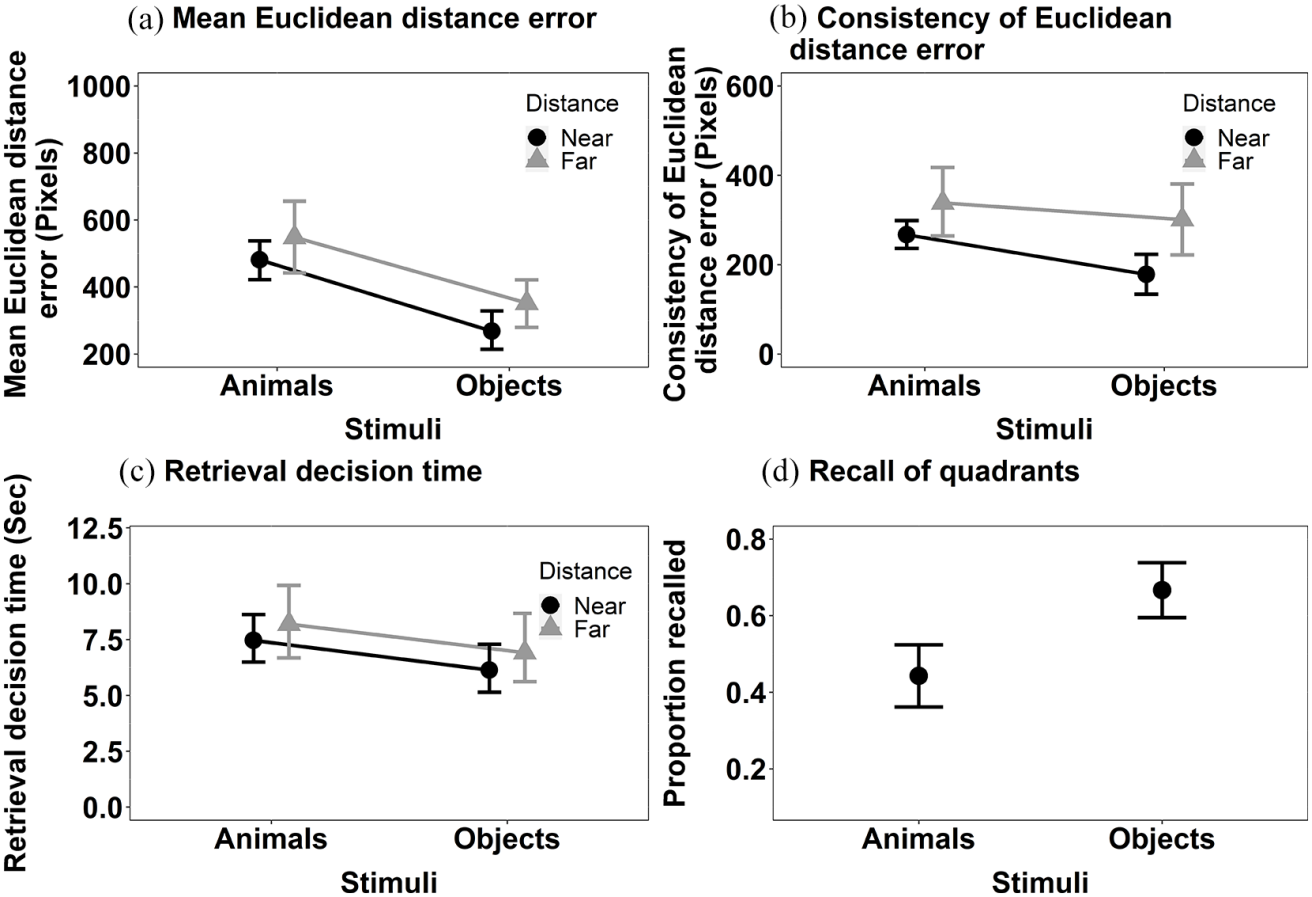
The mean recall performance in Experiment 1b for (a) mean Euclidean distance error for animals and objects in both near and far locations; (b) consistency of Euclidean distance error for animals and objects in both near and far locations; (c) the retrieval decision times for animals and objects in both near and far locations; and (d) proportion of targets correctly recalled in the displayed quadrant. All error bars represent ±95% CI.

*Mean Euclidean distance error* (Figure 4a): The main effect of Stimulus, *F*(1, 34) = 22.39, MSE < 0.001, *p* < .001, η^2^_p_  = .40 was significant but the main effect of Distance was not, *F*(1, 34) = 0.45, MSE < 0.001, *p* = .506, η^2^_p_  = .01. The location of objects was more accurately recalled than that of animals. The interaction between Stimulus and Distance was significant, *F*(1, 34) = 9.33, MSE < 0.001, *p* = .004, η^2^_p_  = .22. Further exploration of the significant interaction between Stimulus and Distance using Bonferroni corrected pairwise comparisons revealed that the effect of Stimulus on the accuracy of decisions about objects held when stimuli were shown near to the centre at encoding, *t*(34) = −5.73, *p* < .001, *d* = −0.003, but just failed to reach significance when decisions were made about stimuli shown far, *t*(34) = −1.95, *p* = .059, *d* = −0.001.

*Consistency of Euclidean distance error* (see Figure 4b): The main effect of Stimulus, *F*(1, 34) = 4.80, MSE = 28,801, *p* = .035, η^2^_p_ = .12, and Distance, *F*(1, 34) = 9.24, MSE = 35,752, *p* = .005, η^2^_p_  = .21, were both significant. The consistency of Euclidean distance error was smaller for objects than animals. The interaction between Stimulus and Distance did not reach significance, *F*(1, 34) = 1.27, MSE = 17,968, *p* = .267,η^2^_p_  = .04.

*Retrieval decision time* (see Figure 4c): The main effect of Stimulus was significant, *F*(1, 34) = 14.60, MSE = 0.002, *p* < .001, η^2^_p_  = .30, but the main effect of Distance was not, *F*(1, 34) = 0.13, MSE = 0.003, *p* = .720, η^2^_p_ < .01 . Decisions in relation to objects were made faster than those for animals. The interaction between Stimulus and Distance, *F*(1, 34) = 0.68, MSE = 0.003, *p* = .416, η^2^_p_  = .02, was not significant.

We also computed the additional analysis of the categorical measure of location, as in Experiment 1a (see Figure 4d). The Wilcoxon signed rank test revealed a significant effect of Stimulus, V = 8, *p* < .001, *r* = .74. The proportion of targets recalled in the correct quadrant was higher for the objects than for animals.

**Figure 5.**
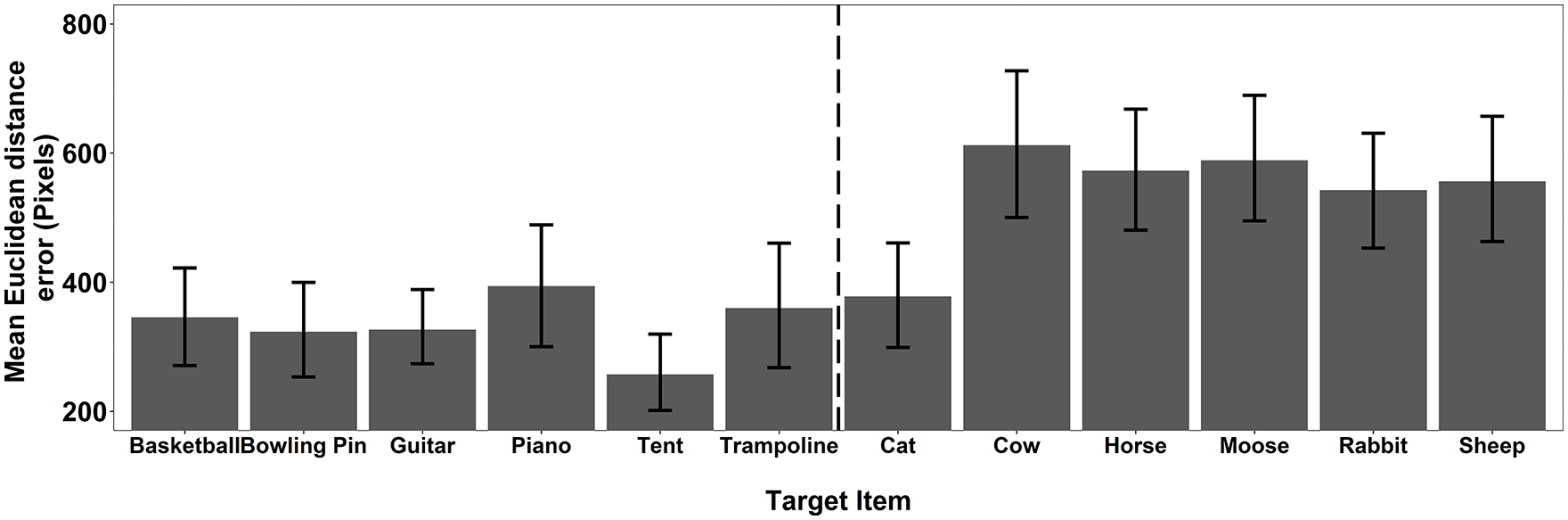
The mean Euclidean distance error for each target item pooled across Experiments 1a and 1b. The error bars represent 95% CI.

As a final stage of the recall analysis, we explored the mean accuracy for each individual target pooled across Experiments 1a and 1b. These data (see Figure 5) predominantly show a consistent story. All but one of the animals show significantly higher consistency of Euclidean distance error than the objects. These data imply that the finding was not simply the result of poor performance with a single animal stimulus.

### Relationship between ratings at encoding and deviation score

**Figure 6.**
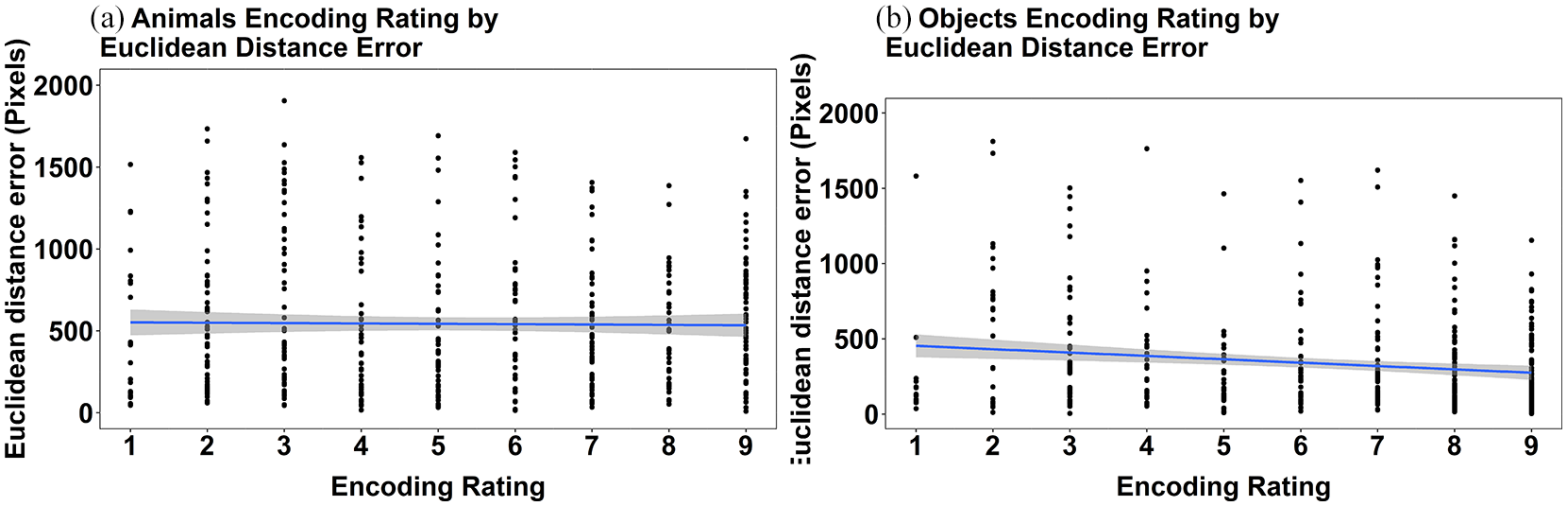
Scatterplots depicting the relationship between ease of collection rating and mean Euclidean distance error for individual targets in the pooled data from Experiments 1a and 1b for (a) animals and (b) objects. The grey bands represent 95% confidence intervals.

Given that the results of Experiment 1b align so closely with those reported for Experiment 1a, we concatenated the datasets and explored the association between the ratings made at encoding and the extent of deviation in memory recall for location. This was done to explore if, at a finer level of analysis than animal and object categories, the perceived ease of collection in the scavenger hunt was associated with error in location recall. These data are presented separately for animals and objects and are shown in Figure 6. The figure shows the relationship between encoding rating and mean Euclidean distance error at the level of individual targets. These data show that there was no significant relationship between encoding rating and mean Euclidean distance error in animals (Rho = −0.03, *p* = .411). In contrast, the data showed a very small but significant negative relationship between encoding rating and mean Euclidean distance error for objects (Rho = −0.13, *p* = .006). Overall, we consider these results to show little to no effect of the encoding rating that is associated with the accuracy of recall performance for animals or objects.

**Figure 7.**
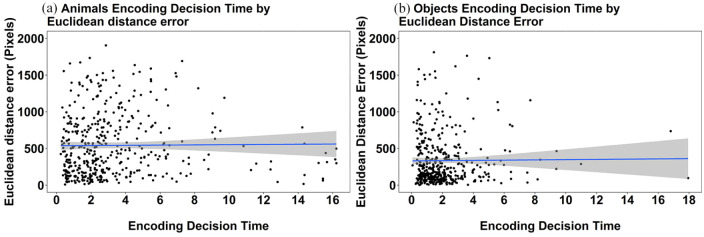
Scatterplots depicting the relationship between encoding decision time and mean Euclidean distance error for individual targets in the pooled Experiments 1a and 1b data for (a) animals only, and (b) objects only. The grey bands represent 95% confidence intervals.

For completeness we also explored the relationship between the encoding decision time and mean Euclidean distance error. These data can be seen in Figure 7. There was a very small though significant relationship in decision time and mean Euclidean distance error in the animals, Rho = 0.123, *p* = .01. With respect to objects, the relationship did not reach significance (Rho = −0.042, *p* = .381). Overall, we consider these results to show little to no effect of the encoding rating that is associated with the encoding decision time of recall for animals or objects.

### Discussion

The results of Experiment 1b confirm those of Experiment 1a. Location memory is more consistent and accurate for objects than animals. Furthermore, the results with respect to the accuracy of location memory held across coordinate and categorical measures of recall. The replication of the poorer recall of the location of animals than objects occurred alongside replication of the findings from Experiment 1a in relation to decision making. Participants took longer to complete the scavenger hunt rating for animals than objects. In addition, participants also took longer to recall the location of animals than they did for objects.

The only finding to differ across Experiments 1a and 1b was the interaction between Stimulus and Distance on the mean Euclidean distance error. The difference in the interactions across the studies is caused by a more marked effect of distance when making decisions about animals presented at encoding in the far location. The difference in findings does not impact the main finding that the recall of animal location is less accurate than that of object recall; it only shows the effect to be magnified in one condition of Experiment 1a. As such we do not consider this interaction further.

The results of the scavenger hunt in both Experiments 1a and 1b showed that participants rated animals as harder to retrieve than objects. However, they also showed that the perceived difficulty of collecting the items in the scavenger hunt was not associated with either error in distance recall or the time to make such a decision. These data support our interpretation of the findings of Experiment 1a.

Nevertheless, the location memory results may be tied to the requirements of the scavenger hunt task in some other way. In Experiment 2 we explore if the results of Experiments 1a and 1b can be replicated using a different encoding task. Participants estimated each of a set of object’s distances (in cm) from the centre of the screen at encoding before later recalling the location of the target objects. If the results of Experiments 1a and 1b were influenced by the specific requirements of the scavenger task, then the results of Experiment 2 should differ from those found in Experiments 1a and 1b. In contrast, if the results of Experiments 1a and 1b are driven by the items themselves, then changing the encoding task should not change the results of the recall location test.

## Experiment 2

### Method

#### Participants

Thirty-one (4M: 27F) individuals who had not participated in Experiment 1a or 1b took part in Experiment 2. They had a mean age of 20.06 years (*SD* = 5.6 years). The sample was again estimated using G*Power 3.1.7 (Faul et al., 2007) using the following parameters: alpha = .05, power = .80, and η^2^_p_  = .06. It was estimated that a sample size of 24 was required to achieve 0.8 power to detect the effect. The sample size was estimated using a medium effect size for the main effect of Stimulus in a 2 × 2 repeated measures ANOVA. The effect size was estimated using the observed accuracy analysis in Experiment 1a, however, using the reported effect size from Experiment 1a (η^2^_p_  = .44) yielded a required sample size of four participants which seemed inappropriate since we had changed the encoding task. Subsequently, we estimated the sample using a reduced η^2^_p_  = .06 value, which represents a more conservative medium effect size.

### Design

The design was identical to that in Experiments 1a and 1b.

### Materials

The only change to the materials was the change in encoding task. Here the scavenger hunt task was replaced by a simple distance estimation task. The task is outlined below.

#### Distance estimation encoding scenario

*In this task, we are interested in how accurately you are able to estimate distance. We are going to show you pictures of a number of items that will be displayed in various locations on the screen. We would like you to estimate how far each item is from the centre of the screen (marked with a black cross) in cm.*

All other stimuli and materials remained the same.

### Procedure

The procedure was predominantly the same as in Experiments 1a and 1b, the only change being exchanging the scavenger hunt encoding task for the distance estimation encoding task. First, participants provided informed consent and then read the distance estimation encoding scenario. They then viewed a target item on the screen for 5 s. On the next screen they were asked to enter how many centimetres away the item was from the centre of the screen. This process was repeated until all of the target items had been viewed. Upon completion of this task, the participants completed the same distractor and retrieval tasks as reported in Experiments 1a and 1b.

### Results

The results were analysed as in Experiments 1a and 1b.

#### Encoding ratings

With respect to distance estimation, the main effect of Distance was significant, *F*(1, 30) = 55.87, MSE = 0.002, *p* < .001, η^2^_p_  = .65, but the main effect of Stimulus did not reach significance, *F*(1, 30) = 1.69, MSE < 0.001, *p* = .204, η^2^_p_  = .05, (see Table 4). Items presented further from the centre at encoding were estimated as such. The interaction between Stimulus and Distance failed to reach significance, *F*(1, 30) = 3.16, MSE < 0.001, *p* = .086, η^2^_p_  = .10.

**Table 4.** The mean distance estimation and encoding decision times in Experiment 2 for both animals and objects stimuli in the near and far encoding tasks. Distances were estimated in centimetres and encoding decision times in seconds.

Distance	Estimated distance (cm)		Encoding decision time (s)	
	Animals	Objects	Animals	Objects
Near	14.99 (8.20)	14.52 (9.48)	5.43 (3.44)	3.22 (1.74)
Far	38.41 (12.64)	41.00 (13.17)	4.98 (2.90)	3.80 (2.00)

With respect to encoding decision time for making distance estimations, the main effect of Stimulus was significant, *F*(1, 30) = 16.25, MSE = 0.01, *p* < .001, η^2^_p_  = .35, but the main effect of Distance did not reach significance, *F*(1, 30) = 3.61, MSE = 0.01, *p* = .067, η^2^_p_  = .11. The interaction between Stimulus and Distance failed to reach significance, *F*(1, 30) = 0.49, MSE = 0.02, *p* = .490, η^2^_p_  = .02.

In sum, the kind of Stimulus influenced the speed of the encoding decision making but not its accuracy. Participants took longer to respond to the animals than objects.

#### Retrieval data

**Figure 8.**
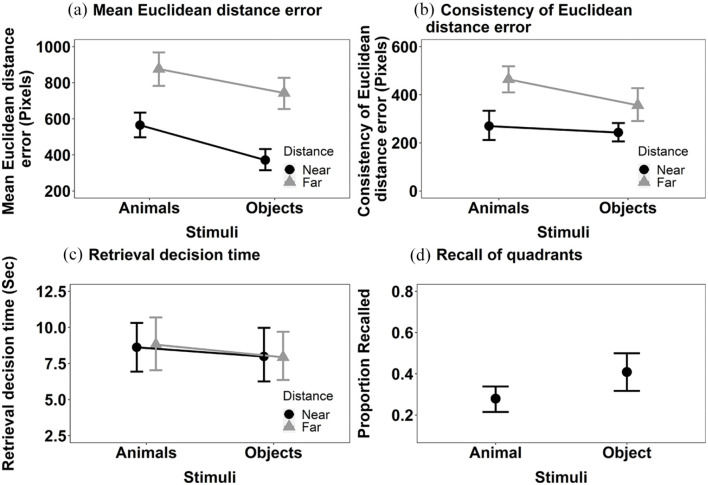
The mean recall performance in Experiment 2 for (a) mean Euclidean distance error for animals and objects in both near and far locations; (b) consistency of Euclidean distance error for animals and objects in both near and far locations; (c) the mean retrieval decision times for animals and objects in both near and far locations; and (d) proportion of targets correctly recalled in the displayed quadrant. All error bars represent ±95% CI.

*Mean Euclidean distance error* (Figure 8a): The main effects of Stimulus, *F*(1, 30) = 15.26, MSE < 0.001, *p* < .001, η^2^_p_  = .34, and Distance, *F*(1, 30) = 42.93, MSE < 0.001, *p* < .001, η^2^_p_  = .59, were significant (see Figure 3b). The location of items was more accurately recalled for objects than animals and if items were presented closer to the centre at encoding than further away. The interaction between Stimulus and Distance did not reach significance, *F*(1, 30) = 4.16, MSE < 0.001, *p* = .050, η^2^_p_  = .12.

*Consistency of Euclidean distance error* (see Figure 8b): The main effects of Stimulus, *F*(1, 30) = 7.35, MSE = 18,982, *p* = .011, η^2^_p_  = .20, and Distance, *F*(1, 30) = 29.33, MSE 25,067, *p* < .001, η^2^_p_  = .49, were significant. The consistency of Euclidean distance error of the recall judgements was higher for animals than objects, and higher for targets that were further away from the centre of the screen than those nearer the centre. The interaction between Stimulus and Distance was not significant, *F*(1, 30) = 1.72, MSE = 29,722, *p* = .200, η^2^_p_  = .05.

*Retrieval decision time* (see Figure 8c): Neither the main effect of Stimulus, *F*(1, 30) = 0.80, MSE = 0.006, *p* = .377, η^2^_p_  = .03, or Distance, *F*(1, 30) = 0.04, MSE = 0.005, *p* = .839, η^2^_p_  < .01, nor their interaction, reached significance, *F*(1, 30) = 0.04, MSE = 0.005, *p* = .843, η^2^_p_  < .01.

We also computed the additional analysis of the categorical measure of location, as in Experiments 1a and 1b. Objects were recalled in the correct quadrant more frequently than animals, V = 60.5, *p* = .03, *r* = .38 (Figure 8c).

### Comparing Experiments 1a, 1b, and 2

We ran one further set of analyses. The consistency of effects reported in each experiment was analysed by mean Euclidean distance error, consistency of Euclidean distance error, and the retrieval decision time across experiments. This was done using a 3 × 2 × 2 mixed ANOVA, with the factors 3(Experiment: Experiments 1a, 1b, and 2), 2 (Stimulus: animals and objects) and 2 (Distance: near and far). The issue of interest is if, and how, the encoding task affected the main effect or interaction; as such we only report the main effects and interactions involving the Experiment factor.

*Mean Euclidean distance error*: The main effect of Experiment was significant *F*(2, 100) = 19.00, MSE = 99,635, *p* < .001, η^2^_p_  = .28. Mean Euclidean distance error was bigger in Experiment 2 than either 1a or 1b. However, a significant two-way interaction between Experiment and Distance, *F*(2, 100) = 12.72, MSE = 46,157, *p* < .001, η^2^_p_  = .20, shows that this was near versus far was significant. The effect of distance was larger in Experiment 2 than either 1a or 1b resulting from markedly bigger error in recalling the location of far stimuli.

The interactions between Experiment and Stimulus, *F*(2, 100) = 0.42, MSE = 51,255, *p* = .655, η^2^_p_  < .01, and Experiment, Stimulus, and Distance, *F*(2, 100) = 1.85, MSE = 42,970, *p* = .162, η^2^_p_  = .04, did not reach significance.

*Consistency of Euclidean distance error*: The main effect of Experiment, *F*(2, 100) = 2.43, MSE = 52,385, *p* = .093, η^2^_p_  = .05, and interactions involving the Experiment factor failed to reach significance; Experiment and Stimulus, *F*(2, 100) = 0.99, MSE = 24,365, *p* = .375, η^2^_p_  = .02, Experiment and Distance, *F*(2, 100) = 0.85, MSE = 34,888, *p* = .430, η^2^_p_  = .02, Experiment, Stimulus, and Distance, *F*(2, 100) = 1.91, MSE = 24,365, *p* = .153, η^2^_p_  = .04).

*Retrieval decision time*: The main effect of Experiment was significant, *F*(2, 100) = 7.44, MSE = 34.11, *p* < .001, η^2^_p_  = .13. Retrieval decision time was longer in Experiment 1a than Experiment 2, *t*(66) = −4.39, *p* < .001, *d* = −2.72, however the contrasts between Experiment 1a and Experiment 1b, *t*(70) = −2.34, *p* = .067, *d* = −1.56, and Experiment 1b and 2, *t*(64) = −1.41, *p* = .498, *d* = −1.16, were not significant. None of the interactions between Experiment and Stimulus, *F*(2, 100) = 0.27, MSE = 9.92 *p* = .766, η^2^_p_  < .01, Experiment and Distance, *F*(2, 100) = 0.39, MSE = 10.18, *p* = .679, η^2^_p_  < .01, Stimulus and Distance, *F*(1, 100) = 0.00, MSE = 11.46, *p* = .984, η^2^_p_ < .01, and, Experiment, Stimulus, and Distance, *F*(2, 100) = 0.03, MSE = 11.46, *p* = .973, η^2^_p_  < .01, reached significance.

### Discussion

Despite the fact the overall recall accuracy is lower in Experiment 2 than either Experiments 1a and 1b, the results across all experiments are strikingly similar. The results of Experiment 2 show the location of objects to be recalled more accurately than those of animals as indicated by the analysis of mean Euclidean distance error. In addition, participants were faster to estimate the distance of objects from the centre of the screen than that of animals. The results of Experiment 2 provide evidence that the poor accuracy of recall for the location memory of animals relative to objects reported in Experiments 1a and 1b did not result from the scavenger encoding task.

The cross-experiment analysis shows Experiment 2 to have markedly affected the accuracy of recall of stimuli, particularly those presented in the far locations. There was also evidence, at least with respect to Experiment 1a, that the retrieval decision time was longer when the scavenger hunt encoding task was used rather than the distance estimation encoding task. However, most importantly for the present study, there was no evidence of an interaction involving both Experiment and Stimulus. The analysis provides evidence that the effect of stimulus type on location recall is generalised across encoding tasks.

Despite evidence of a modest influence of the encoding task on performance across Experiments 1a, 1b, and 2, the striking consistency in the pattern of findings leads us towards the conclusion that a difference in how animals and objects are attended at encoding impacts the accuracy of location recall. These data are consistent with the explanation given in Experiment 1a. We now consider this issue in the “General discussion” section.

## General discussion

The present study reports three experiments exploring location memory for images of animals and objects. Taken together, the results show that the location of objects is recalled more accurately and faster than the location of animals. The results were found irrespective of the encoding task used showing that the results are unlikely to be an artefact of the specific encoding task used when first viewing the items. In addition, the analysis presented in Experiment 1a, allied to our control across the sets of animals and objects for familiarity, visual complexity, object agreement, viewpoint, and manipulability, rule these out as confounding variables. Consistent with one of the hypotheses outlined in the “Introduction”, we suggest that the data are best explained as a consequence of images of animals activating late selection object-based attention such that their representation leads to a loss in the precision of location memory.

In the current study, we have avoided using the term the “Animacy effect,” however, it is possible that some functional attributes of animals contribute to the poor encoding of location. For instance, animals by definition, are mobile and move of their own volition. As such, since an animal’s location is likely to change, there may be reduced value to encoding an animal’s location. Indeed, it may be that the mobile nature of animals encourages animals to be selected late by object-based attention. This explanation is consistent with the Animacy Effect as observed with word recall (e.g., Gelin et al., 2017; Nairne et al., 2017; Nairne et al., 2013).

If the explanation we have offered is correct then the present results extend those studies that have explored the impact of kinds of object images on attention (e.g., Guerrero & Calvillo, 2016; Hu et al., 2023). It is important, however, to note that we did not take a measure of object-based attention in the present study. Having shown that there is an increased error in location recall for animals relative to objects, a future study should explore the direct association between object-based attention and error of location memory.

There may be alternative explanations for the findings of the present study. The first alternative explanation we offer is that the visual similarity between exemplars within each category may increase interference in the recall of the location of animals relative to objects. The stimuli in the current experiments were chosen from the BOSS stimulus database and were controlled along a number of dimensions, including familiarity and object agreement. However, animals are likely to form a more homogeneous group than objects (for instance, all the animals were four-legged, with a number being similar in colour). It might be, therefore, that location recall errors are linked to category homogeneity. A challenge to this account is that the stimuli were all readily identifiable and easily named (a post hoc check using the BOSS stimuli normative data [Brodeur et al., 2014] show comparable scores in naming agreement for the two categories). As such, interference affecting location recall would have to coexist with ready identification and naming. It seems unlikely to be the case, but we cannot rule this explanation out in the current series of experiments. It is important for future work to explore this issue.

The second alternative explanation we offer relates to the minimal context in which items were viewed. Scene contexts are known to be important to item detection (Biederman, 1972) and to guide attention (in fact eye movements) to likely target locations (Neider & Zelinsky, 2006). More specifically to the present situation, it may be that, for some reason, the recall of animals is affected more than that of objects by the absence of contextual information at encoding and recall, analogous to the way culture has been suggested to influence attention to contexts (Masuda & Nisbett, 2001). The present data cannot test this account and we know of no relevant, previously reported data. However, the effect on the recall of animals and objects of presenting contextual information at encoding and recall is worthy of exploration.

A final possible explanation we offer of the results reported is that the difference in recall of the location of animals and objects reflects a systematic response artefact. For example, participants might have considered the centre of an animal to be shifted towards its head rather than its body. We can, however, provide some relevant data in relation to this account. All images were 80 × 80 pixels square, so the maximum possible deviation within an image is 113 pixels (measured across the diagonal). Therefore, if the observed difference was the result of a systematic difference in what was considered the centre of animals versus objects, then the difference would have to be less than 113 pixels. Across Experiments 1–3, the mean difference in deviation for animals and objects was 194 pixels. As such, while differences in the variability of what is considered the centre of animals and objects may contribute to the effects we report, they cannot account for them in their entirety.

There are limitations to the current study. There may be a concern over the number of items used to test location memory as generalising to a broad category from six examples of that category is potentially problematic. Nevertheless, previous studies of location memory that have used the scavenger hunt paradigm (Clark & Bruno, 2016; Nairne et al., 2012) to explore location memory in a survival processing paradigm have used fewer items (8) than the present study. Moreover, in the wider spatial memory literature where memory for items or item images has been tested, it is common to have comparatively small sets of stimuli (e.g., fewer than 15 items—Baguley et al., 2006; Choi & L’Hirondelle, 2005; Clark et al., 2013; Postma et al., 1998; Siegel & Castel, 2018; Shih et al., 2012). Thus, the small number of items shown in the present study is not atypical for memory studies. Studies exploring attention to pictorial items typically have high numbers of trials but with repetitions of the small set of items. For example, the number of target images used in the studies of Guerrero and Calvillo (2016) and Hu et al. (2023) ranged from 4 to 12. In sum, although we recognise that participants viewed a small number of items in the present study, and see a risk in that fact for generalising the findings, the number is in line with prior literature. It should, however, be noted that some spatial memory studies have employed larger stimulus sets (though commonly in intentional paradigms, e.g., Harlow & Donaldson, 2013; Harlow & Yonelinas, 2016; Nilakantan et al., 2018) and the impact of this should be explored in future work.

A further limitation to acknowledge is that while we report data from three separate studies, we acknowledge that our sample was limited to students from a UK university. There have been concerns raised in the literature regarding the ability of studies with limited demographic ranges to generalise to a wider population (e.g., Barrett, 2020a, 2020b; Pollet & Saxton, 2019; Simons et al., 2017). While the findings reported are consistent, we cannot generalise this finding to a wider population. We do not believe that this invalidates the findings, but it does encourage the testing of a broader population.

In conclusion, the present study explored the recall of the location of animals and objects in two incidental encoding tasks. The results demonstrated that the location of objects is recalled more accurately and faster than that of animals. We suggest that the findings result from increased object-based attention to animals relative to objects leading to recall uncertainty of the specific location of item presentation shown at encoding.

## Notes

1. Gelin et al.’s study followed on from a significant body of experimental literature showing how animacy affects word memory (the animacy effect [AE]; Blunt & VanArsdall, 2021; Bonin et al., 2014, 2015, 2022; Félix et al., 2019; Gelin et al., 2017, 2018, 2019; Kazanas et al., 2020; Komar et al., 2023; Leding, 2019, 2020; Madan, 2021; Nairne et al., 2013, 2017; Popp & Serra, 2016, 2018; Serra, 2021; VanArsdall et al., 2017; van Buren & Scholl, 2017). The typical finding in this literature is that item recall is better for words naming animals than objects.

2. While we are primarily interested in the main effect, we note that a post hoc sensitivity test revealed that the interaction test for a η^2^p = .06 would achieve power = .63.

3. In the current dataset, the majority of normality tests (using Shapiro–Wilk) revealed that the data could be considered normally distributed, however, there were a number of outliers which could potentially impact our results. The reciprocal transformation is computed as 1/raw score, this allows all data to be analysed while limiting the potential influence of any extreme outlying data points. The reciprocal transformation is effective when there are large outliers in the positive tail of the distribution (Baguley, 2018; Field, 2013; Howell, 2012). All data reported in the tables and figures are generated using the original data and not data which have been through a reciprocal transformation.
